# Immunization with whole cell but not acellular pertussis vaccines primes CD4 T_RM_ cells that sustain protective immunity against nasal colonization with *Bordetella pertussis*

**DOI:** 10.1080/22221751.2018.1564630

**Published:** 2019-01-21

**Authors:** Mieszko M. Wilk, Lisa Borkner, Alicja Misiak, Lucy Curham, Aideen C. Allen, Kingston H. G. Mills

**Affiliations:** Immune Regulation Research Group, School of Biochemistry and Immunology, Trinity Biomedical Sciences Institute, Trinity College Dublin, Dublin, Ireland

**Keywords:** *Bordetella pertussis*, pertussis vaccine, tissue-resident memory T cell, Th1 and Th17 cells

## Abstract

Protective immunity wanes rapidly after immunization of children with acellular pertussis (aP) vaccines and these vaccines do not prevent nasal colonization or transmission of *Bordetella pertussis* in baboons. In this study, we examined the role of tissue-resident memory T (T_RM_) cells in persistent protective immunity induced by infection or immunization with aP and whole-cell pertussis (wP) vaccines in mice. Immunization of mice with a wP vaccine protected against lung and nasal colonization, whereas an aP vaccine failed to protect in the nose. IL-17 and IFN-γ-secreting CD69^+^CD4^+^ T_RM_ cells were expanded in the lung and nasal tissue after *B. pertussis* challenge of mice immunized with wP, but not aP vaccines. However, previous infection induced the most persistent protection against nasal colonization and this correlated with potent induction of nasal tissue T_RM_ cells, especially IL-17-secreting T_RM_ cells. Blocking T cell migration to respiratory tissue during immunization with a wP vaccine impaired bacterial clearance, whereas transfer of T_RM_ cells from convalescent or wP-immunized mice conferred protection to naïve mice. Our findings reveal that previous infection or wP vaccination are significantly more effective than aP vaccination in conferring persistent protective immunity against *B. pertussis* and that this is mediated by respiratory T_RM_ cells.

## Introduction

Whooping cough (pertussis) is a vaccine-preventable infectious disease caused by the bacterium *Bordetella pertussis*. Pertussis was a well-controlled respiratory disease following decades of use of whole-cell pertussis (wP) vaccines. However, concerns around the safety of wP vaccines led to their replacement with acellular pertussis (aP) vaccines in most developed countries in the 1990s–2000s. Despite high vaccine coverage, pertussis is now a re-emerging infectious disease in many countries [1,2].

The resurgence of pertussis has been attributed to a number of different factors. The emergence of *B. pertussis* strains with deletions or mutations in pertussis toxin (PT) and pertactin (PRN), key protective antigens in the aP vaccine, may have resulted in escape from protective immunity induced with aP vaccines [3,4]. However, immune driven antigen variation is less of an issue with the wP vaccine, because of the broad range of potentially protective antigens in this vaccine. The resurgence of whooping cough may also reflect improved diagnosis and reporting of cases of pertussis [5]. However, there have also been a significant number of infant deaths from pertussis in countries with high aP vaccine coverage [6]. While most of these have been in infants under 3 months of age [6] and might have been prevented by maternal immunization [7], this also points to a failure of the aP vaccine-induced immunity to prevent transmission of *B. pertussis* in the community.

Immunization of infants and children with aP vaccines induces potent antibody responses specific for the vaccine antigens detectable by ELISA [8,9]. While there have been some suggestions from household contact studies that the levels of antibodies against PT, PRN or fimbriae may correlate with protection against disease [10,11], it is not clear if antibodies against these antigens can prevent infection with *B. pertussis* [8,9]. Studies on cellular immune responses in humans have demonstrated that *B. pertussis*-specific Th1 responses are induced by *B. pertussis* infection or immunization with wP vaccines, whereas aP vaccines predominantly induce Th2-type responses [12–14]. Consistent with these findings, studies in a mouse model have shown that aP vaccines induce Th2-polarized responses and weak Th17 responses, but undetectable Th1 responses [15]. In contrast, wP vaccines and natural infection induce potent Th1 and Th17 responses and confer higher protection against lung infection of mice with *B. pertussis* [15,16].

Most of the studies to date on vaccine-induced protective immunity in mouse models have focused on preventing lung infection and have not examined the impact of immunization on colonization of the nose. Studies in a baboon model demonstrated that previous infection, and to a lesser extent immunization with a wP vaccine, prevented nasal colonization, whereas immunization with an aP vaccine did not prevent nasal colonization or transmission to naïve baboons [17]. There is also indirect evidence in humans of asymptomatic transmission of *B. pertussis* from aP-vaccinated to naïve individuals [18]. Thus, while aP vaccines may be capable of preventing severe disease in a high proportion of vaccinated individuals for a finite time period after vaccination, they may not prevent nasal colonization and transmission of *B. pertussis* in humans.

It has also been demonstrated that immunity wanes rapidly after immunization of infants with aP vaccines [19]. A study in the US reported that the effectiveness of an aP vaccine was 41% and 24% for 2–7- and 8–12-year-olds, respectively [20]. Another study estimated that only 10% of children would be immune 8.5 years after the last dose of DTaP [21]. The durability of protective immunity was greater in recipients of one or more doses of a wP vaccine compared with a full course of aP vaccines [22,23]. Evidence is emerging that T and B cell memory, which sustain protective immunity, may be more persistent after immunization with wP compared with aP vaccines [24]. Furthermore, priming and boosting with an aP vaccine failed to generate memory Th1 and Th17 cells, whereas priming with a wP vaccine generated persistent *B. pertussis*-specific Th1 and Th17 responses, even in individuals boosted several times with an aP vaccine [25]. Memory T cells that reside in mucosal tissue during and after infection, termed tissue-resident memory T cells (T_RM_ cells), are now considered to play a critical role in the rapid clearance of viruses and bacteria after re-infection with the same pathogen [26–28]. We have shown that infection of naïve mice with *B. pertussis* induces CD4 T_RM_ cells that are maintained in the lung after bacterial clearance. These CD4 T_RM_ cells expand rapidly after re-infection with *B. pertussis* and mediate rapid clearance of bacteria from the respiratory tract [29].

In this study, we have examined the capacity of wP and aP vaccines to induce T_RM_ cells and to protect against nasal colonization of mice with *B. pertussis*, and compared this with immunity induced by previous infection. Our findings demonstrate that while immunization of mice with an aP vaccine failed to generate CD4 T_RM_ cells or to protect against nasal colonization, immunization with a wP vaccine primed IL-17- and IFN-γ-secreting CD4 T_RM_ cells in the lungs and nasal tissue, and prevented nasal colonization as well as lung infection with *B. pertussis*. However, the most persistent protection was induced by natural infection and this reflected the most potent induction of T_RM_ cells, especially IL-17-secreting T_RM_ cells.

## Results

### Immunization with a wP, but not an aP, vaccine induces T_RM_ cells and protects against nasal colonization with B. pertussis

**Figure 1.**
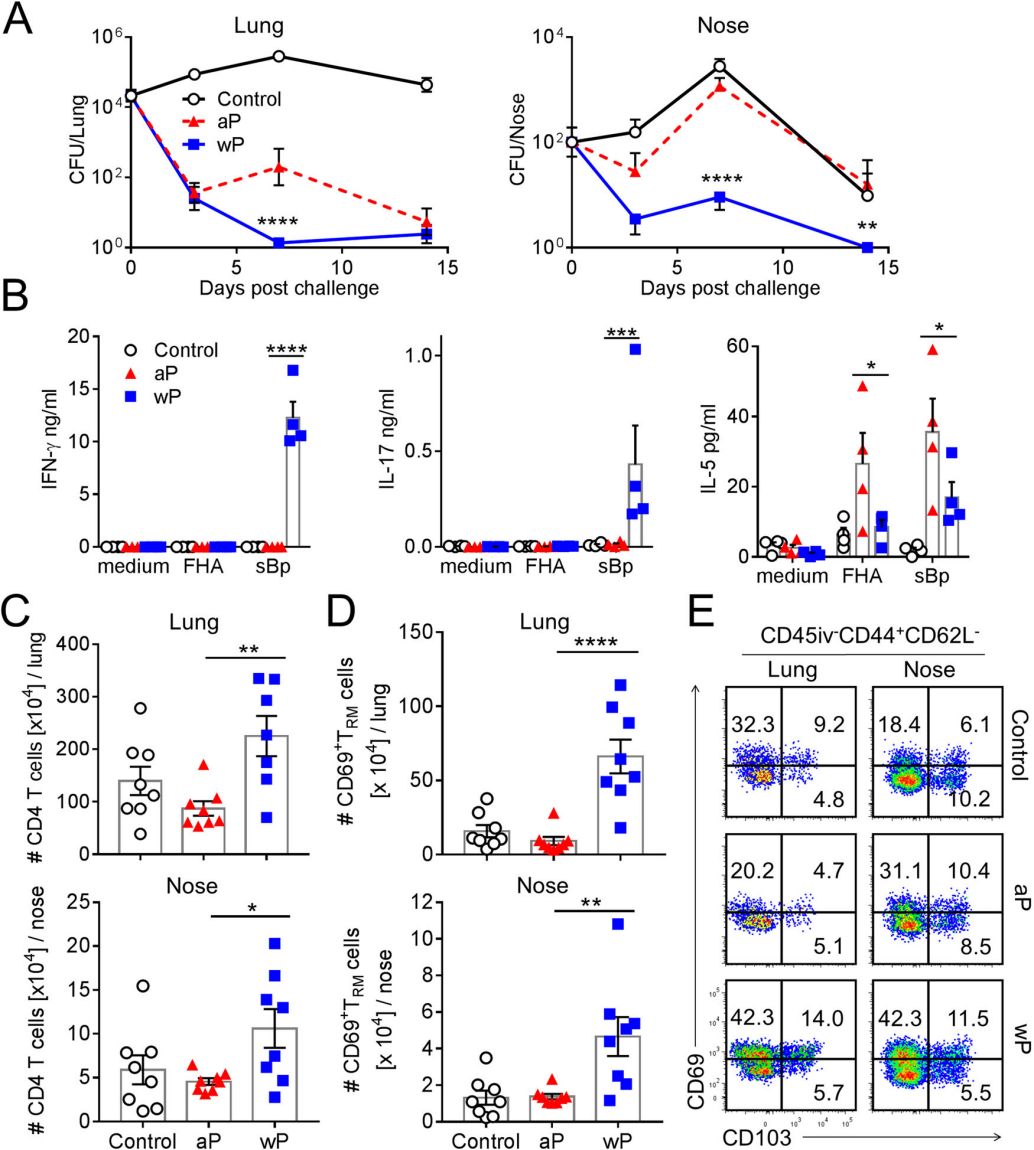
Immunization with a wP vaccine promotes formation of CD69^+^CD4^+^ T_RM_ cells in the nose and lungs and prevents nasal colonization. C57BL/6 mice were immunized with 1/50 human dose of aP (Boostrix) or wP (NIBSC) twice (-6 and -2 weeks) before aerosol challenge with *B. pertussis*. (a) CFUs in the lungs and nose were enumerated at indicated time points. Lung and nose CFU counts (mean ± SEM *n* = 8 mice per group per time-point in two independent experiments). **p < .01, ****p < .0001 versus aP by two-way ANOVA with the Tukey’s post-test. (b) On the day of but prior to *B. pertussis* challenge spleen cells were stimulated with FHA or sonicated *B. pertussis* (sBp) or medium only and IFN-γ, IL-17 and IL-5 concentrations in supernatants were quantified by ELISA after 3 days of culture. Results shown are mean ± SEM (*n* = 4 mice in triplicate). *p < .05, ***p < .001, ****p < .0001 versus aP by one-way ANOVA with the Tukey’s post-test, only significant differences between experimental groups are indicated. (c–e) Seven days post challenge, mice were injected i.v. with anti-CD45 Ab 10 min prior to euthanasia to discriminate circulating from tissue-resident leukocytes by flow cytometry. (c) Total number of CD4 T cells in the lungs (upper graph) and nasal cavity (lower graph). (d) Total number of CD45iv^–^CD44^+^CD62L^–^CD69^+^CD4^+^ T_RM_ cells in the lungs and nasal cavity. Results (c and d) are mean ± SEM (*n* = 8 mice in two independent experiments) *p < .05, **p < .01, ****p < .0001 versus aP by one-way ANOVA with the Tukey’s post-test, only significant differences between experimental groups are indicated. (e) Representative plots for expression of CD69 and CD103 on CD45iv^–^CD44^+^CD62L^–^CD4^+^ T cells.

We examined the relative ability of aP and wP vaccines to confer protective immunity in the upper and lower respiratory tracts and to induce T_RM_ cells. Mice were immunized 6 and 2 weeks before aerosol challenge with live *B. pertussis*. Mice immunized with a wP vaccine rapidly cleared the infection from the lungs, whereas mice immunized with an aP vaccine had delayed clearance, with a significantly higher bacterial count 7 days post-infection when compared with mice immunized with the wP vaccine (Figure 1(a)). Strikingly, while wP-immunized mice controlled the infection in the nasal cavity, rapidly reducing bacterial counts by day 3 and completely clearing the infection by day 14 post challenge, aP vaccinated mice had not cleared the bacteria from the nose by day 14 post challenge and had similar bacterial counts to the unimmunized control mice (Figure 1(a)).

We assessed antigen-specific immune responses induced with aP and wP vaccines by stimulation of spleen cells from immunized mice with *B. pertussis* antigens. We found that *B. pertussis*-specific IFN-γ and IL-17 was produced by spleen cells from mice immunized with the wP vaccine (Figure 1(b)). In contrast, spleen cells from mice immunized with the aP vaccine produced IL-5, but not IFN-γ or IL-17, in response to sonicated *B. pertussis* (sBp) and FHA. These findings are consistent with previous reports that wP vaccines generate Th1 and Th17 cells, whereas aP vaccines preferentially induce Th2 responses [15].

Analysis of immune cells in the lung and nasal tissues revealed that mice immunized with a wP vaccine had significantly more CD4 T cells in both tissues when compared to aP-immunized mice 7 days post challenge (Figure 1(c)). In contrast, the numbers of CD8 T cells and B cells in lung and nasal tissue were not enhanced in immunized mice (Supplementary Figure 1(a)).

We characterized tissue-resident CD4 T cells in the upper and lower respiratory tracts using a validated intravenous labelling approach to discriminate circulating (CD45iv^+^) from tissue-resident (CD45iv^-^) cells, together with staining for CD69 and CD103, markers expressed on some but not all T_RM_ cells [28]. T cells that are resident in the tissues (e.g. lungs or nasal tissue) are termed “tissue-resident T cells”, and are defined through lack of *in vivo* labelling with anti-CD45 antibody (CD45iv^-^) after intravenous (i.v.) injection of mice 10 min prior to euthanasia. Within this CD45iv^-^ population, cells that are CD44^+^CD62L^-^ and express CD69, with or without CD103, are considered to be “tissue-resident memory T cells”.

The results revealed that lung tissue, which is highly vascularized compared with the nasal tissue, contained a large fraction of circulating CD45iv^+^CD4^+^ T cells (Supplementary Figure. 1(b)). In contrast, the majority of nasal tissue CD4 T cells were protected from i.v. labelling, suggesting that this tissue is less accessible for circulating cells (Supplementary Figure 1b). Mice immunized with the wP vaccine had significantly higher numbers of CD69^+^CD4^+^ T_RM_ cells in the lungs when compared with aP-immunized mice 7 days post challenge and a small proportion of these were CD69^+^CD103^+^CD4^+^ T_RM_ cells (Figure 1(d,e)). While the frequency of total CD4 T cells was not substantially different in the nasal cavity of mice immunized with aP or wP vaccines, the number of total CD4 T cells was significantly higher in the nasal cavity of mice immunized with the wP vaccine (Figure 1(c), supplementary Figure 1(c)) and immunization with the wP vaccine also induced significantly greater frequencies and numbers of CD69^+^CD4^+^ T_RM_ cells in the nose when compared with mice immunized with the aP vaccine (Figure 1(d), supplementary Figure 1(c)). Collectively, these findings demonstrate that the wP vaccine induced superior protective immunity than the aP vaccine, especially in the nasal cavity, and that the wP, but not the aP, vaccine generated CD4 T_RM_ cells in the upper and lower respiratory tracts.

### CD4 T_RM_ cells induced in the lung and nasal tissue by immunization with a wP vaccine potently secrete IFN-γ and IL-17

**Figure 2.**
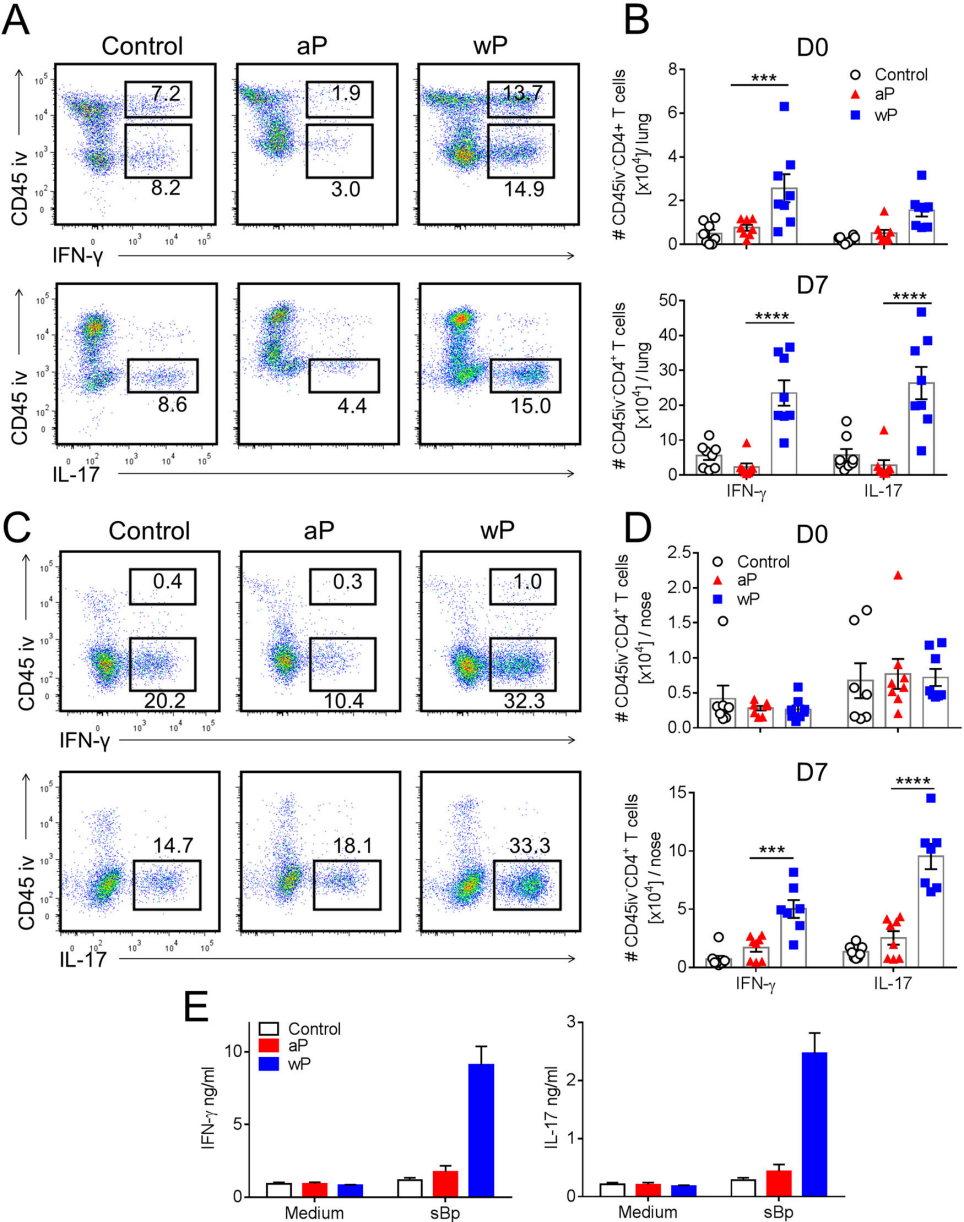
CD4 T cells in the lung and nasal tissue of mice immunized with a wP vaccine and challenged with *B. pertussis* potently secreted IFN-γ and IL-17. C57BL/6 mice were immunized and challenged, and tissue-resident cells were quantified as described in Figure 1. Intracellular cytokine staining and flow cytometry were performed on lung and nasal tissue T cells one day before (D0) and 7 days post challenge (D7). Representative plots showing IFN-γ and IL-17 secretion by lung (a) and nasal tissue (c) CD4 T cells. Total number of IFN-γ- and IL-17-secreting lung and nasal CD45iv^–^CD4 T cells in the lungs (b) and nasal tissue (d). Results shown are mean ± SEM (*n* = 8 in two independent experiments). ***p < .001, ****p < .0001 versus aP by two-way ANOVA with the Tukey’s post-test, only significant differences between experimental groups are indicated. (e) Tissue-resident CD45iv^–^CD4^+^ T cells purified from the lungs 2 weeks post immunization (prior to *B. pertussis* challenge) were stimulated with sonicated *B. pertussis* (sBp) in the presence of irradiated splenic APC. After 3 days of culture, IFN-γ and IL-17 was quantified in supernatants by ELISA. Results shown are mean ± SD for triplicate culture of cells pooled from 4 mice per group.

Studies in humans and mice have shown that wP vaccines induce *B. pertussis*-specific Th1 and Th17 responses, whereas aP vaccines preferentially induce Th2 responses, detectable in the blood or spleen [13,15,25]. However, there is less information available on T cell responses at the site of infection in the respiratory tract. Here we performed intracellular staining (ICS) and flow cytometry on the cells isolated from the lung and nasal tissues of mice immunized with aP or wP vaccines. Intravenous staining of circulating leukocytes was performed to discriminate circulating (CD45iv^+^) from tissue-resident (CD45iv^-^) CD4 T cells. Interestingly, IFN-γ was secreted by both circulating and tissue CD4 T cells, whereas IL-17-secreting CD4 T cells were primarily localized in the lung tissue (Figure 2(a)). Lung-resident CD4 T cells from wP-immunized mice secreted significantly more IFN-γ and IL-17 than lung-resident CD4 T cells from aP-immunized mice on the day of challenge (Figure 2(b)). Following *B. pertussis* challenge (day 7), the frequency and number of IFN-γ- and IL-17-producing CD45iv^–^CD4^+^ T cells increased in the lungs of mice immunized with a wP vaccine, whereas the numbers of Th1- and Th17-type CD4 T cells in aP-immunized mice were below the background level seen in control non-immunized mice (Figure 2(a,b) and supplementary Figure 1(d)).

CD4 T cells from the nasal cavity were mainly CD45iv^-^ tissue-resident T cells and a proportion of these secreted IFN-γ and IL-17 (Figure 2(c)). Interestingly, before *B. pertussis* challenge there was a relatively low number of CD4 T cells in the nasal tissue producing only background levels of cytokines. However, IFN-γ- and IL-17-producing CD4 T cells were recruited to or expanded in the nasal tissue of wP-immunized mice 7 days post *B. pertussis* challenge, and the frequency and numbers were significantly greater than in aP-immunized or non-immunized mice (Figure 2(d), supplementary Figure 1(d)).

In order to demonstrate that at least a proportion of the lung T cells infiltrating the respiratory tract were *B. pertussis*-specific, we purified CD45iv^–^CD4^+^ T cells from the lungs 2 weeks post immunization, and stimulated them with sonicated *B. pertussis* in the presence of antigen-presenting cells (APC). The data revealed potent IFN-γ and IL-17 production by tissue-resident CD4 T cells from the lungs of mice immunized with the wP but not the aP vaccine (Figure 2(e)). We could not detect antigen-specific IL-5 production by lung tissue-resident CD4 T cells (data not shown). These findings demonstrate that parenteral immunization with a wP vaccine generates *B. pertussis*-specific T cells that migrate to the lungs prior to challenge with *B. pertussis*.

**Figure 3.**
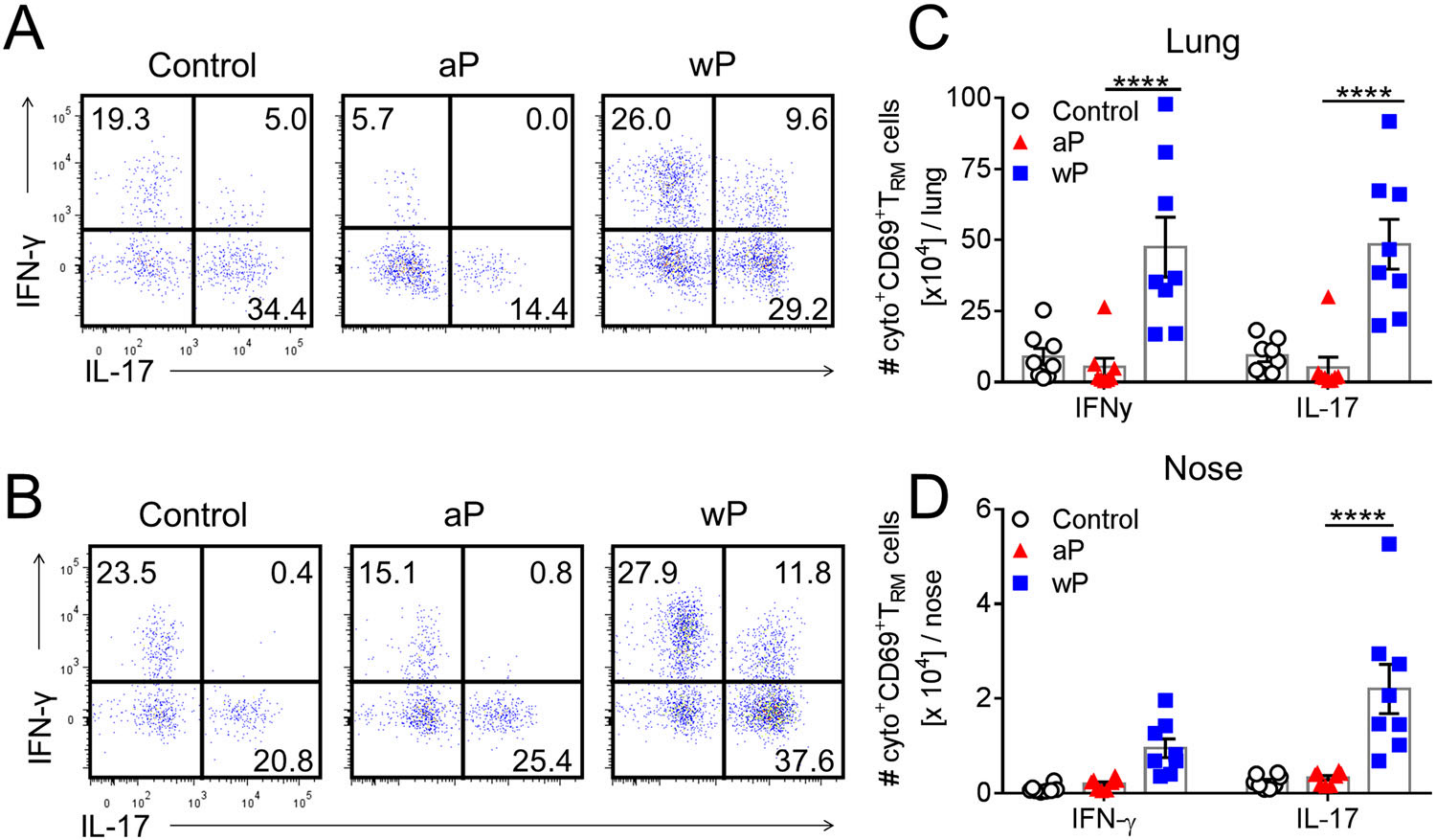
CD69^+^CD4^+^ T_RM_ cells induced in the lungs and nasal tissue by immunization with a wP vaccine potently secreted IFN-γ and IL-17. C57BL/6 mice were immunized and challenged, and tissue-resident cells were quantified as described in Figure 1. Representative FACS plots and total number of IFN-γ- and IL-17-secreting CD69^+^CD4^+^ T_RM_ cells in the lungs (a) and nasal cavity (b) 7 days post challenge. (c, d) Absolute numbers of IFN-γ or IL-17-producing CD69^+^CD4^+^ T_RM_ cells in the lung (c) or nasal tissue (b). Results shown are mean ± SEM (*n* = 8 mice in two independent experiments). ****p < .0001 versus aP by two-way ANOVA with the Tukey’s post-test, only significant differences between experimental groups are indicated.

We next analysed the production of Th1- and Th17-type cytokines by lung and nasal CD69^+^ T_RM_ cells in immunized mice 7 days post *B. pertussis* challenge. CD69^+^CD4^+^ T_RM_ cells in the lungs and nasal tissue of mice immunized with a wP vaccine secreted IFN-γ, IL-17 or co-produced both cytokines (Figure 3(a,b)). In contrast, CD69^+^CD4^+^ T_RM_ cells that produced IFN-γ or co-produced IFN-γ and IL-17 were at or below background levels in mice immunized with an aP vaccine (Figure 3(a,b)). The absolute numbers of IFN-γ- and IL-17-producing CD69^+^CD4^+^ T_RM_ cells in the lungs were significantly higher in wP-immunized, compared with aP-immunized mice (Figure 3(c)). In the nasal tissue there was significantly more IL-17-producing CD69^+^CD4^+^ T_RM_ cells in wP-immunized compared with aP-immunized mice (Figure 3(d)). The absolute number of IFN-γ-secreting CD69^+^CD4^+^ T_RM_ cells was also elevated, though not significantly, in wP-immunized mice. Our findings revealed that unlike the aP vaccine, the wP vaccine was a potent inducer of Th1- and Th17- type immune responses locally in the lung and nasal tissue. Furthermore, the wP vaccine promoted the formation of CD69^+^CD4^+^ T_RM_ cells that co-secreted IFN-γ and IL-17 in the upper and lower respiratory tracts. This suggests that wP, but not aP, vaccines induce respiratory CD69^+^CD4^+^ T_RM_ cells with polyfunctional properties.

### Sustained protective immunity against nasal colonization induced by previous infection or immunization with a wP vaccine is associated with the induction of T_RM_ cells

**Figure 4.**
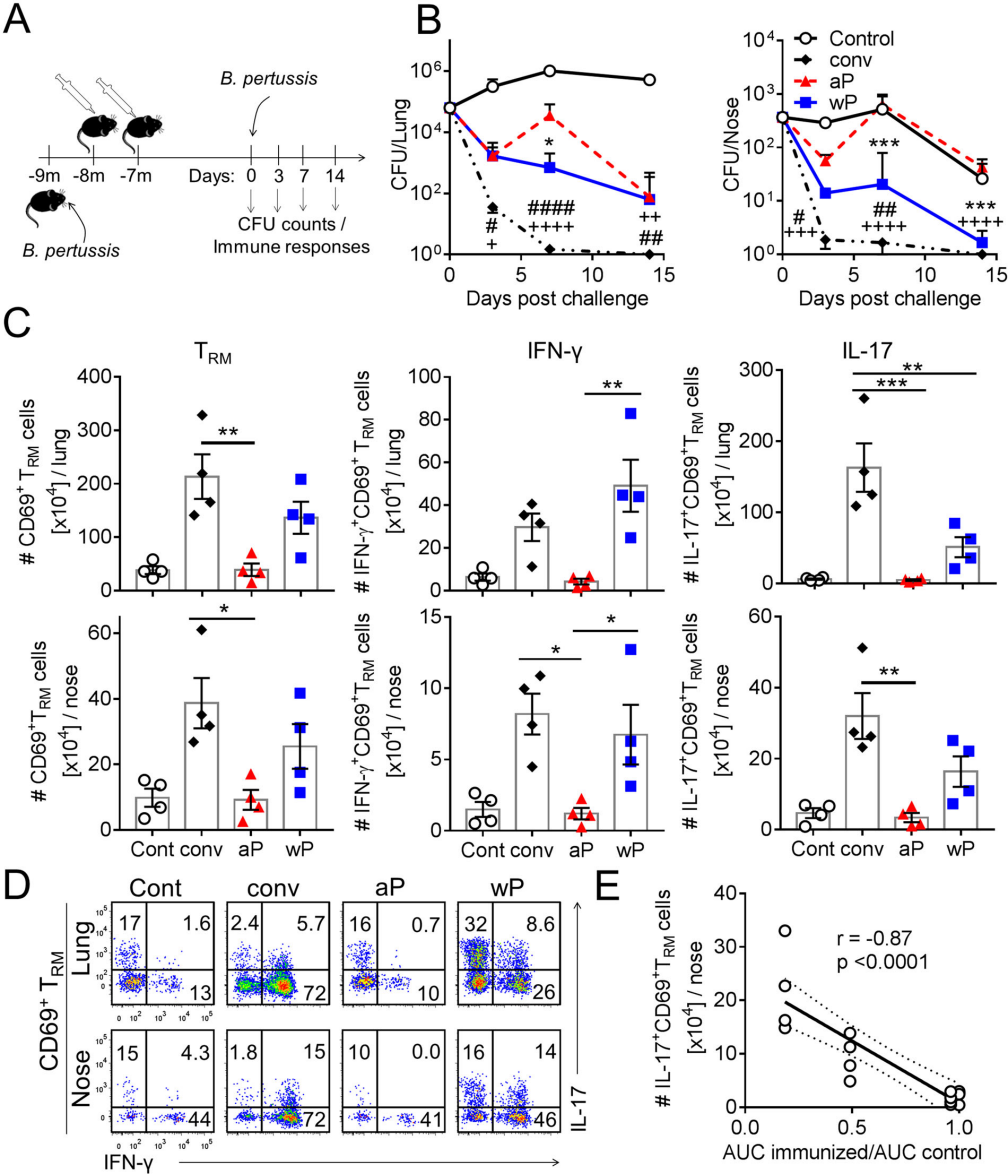
Sustained protective immunity against nasal colonization induced by previous infection or immunization with a wP vaccine correlates with the induction of T_RM_ cells. (a) Schematic of immunization and challenge protocol. C57BL/6 mice were either aerosol infected with *B. pertussis* (conv), or immunized with 1/50 human dose of aP (Boostrix) or wP (NIBSC) twice at an interval of 4 weeks. Seven months after clearance of primary infection or after the 2nd immunization, the animals were aerosol challenged with *B. pertussis.* (b) CFUs in lung and nasal tissue homogenates were determined 3, 7, and 14 days post challenge. Results are mean ± SEM *n* = 4 mice per group per time-point. *p < .05, ***p < .001 aP versus wP; ^#^*p* < .05, ^##^*p* < .01, ^####^*p* < .0001 wP versus conv; ^++^*p* < .01, ^+++^*p* < .001, ^++++^*p* < .0001 aP versus conv by two-way ANOVA with the Tukey’s post-test. (c) Seven days post challenge, lymphocytes from the lungs and nasal tissue were prepared by digestion. Prior to euthanasia, the animals were i.v. injected with fluorochrome-coupled CD45 antibody to allow the discrimination of tissue-resident and circulating cells. Results are mean ± SEM (*n* = 4 mice) absolute numbers of CD69^+^CD4^+^ T_RM_ cells (left graphs), IFN-γ-producing CD69^+^ CD4^+^ T_RM_ cells (middle graphs) and IL-17-producing CD69^+^CD4^+^ T_RM_ cells (right graphs) in the lung (top) or nasal tissue (bottom). *p < .05, **p < .01, ***p < .001 by one-way ANOVA with the Tukey’s post-test, only significant differences between experimental groups are indicated. (d) Representative plots showing secretion of IFN-γ and IL-17 by CD45iv^–^CD44^+^CD62L^–^CD69^+^CD4^+^ T cells. (e) Correlation between protection against nasal colonization and the absolute number of IL-17-producing CD69^+^CD4^+^ T_RM_ cells in that tissue for the corresponding mouse. Protection was expressed as a ratio of the area under the curve (AUC) of bacterial clearance (day 0–14) after challenge of immunized versus the AUC for control unimmunized mice, as previously described [45]. Dotted lines indicate 95% confidence bands of the best fit line.

It is well established that protective immunity wanes rapidly after immunization of infants and children with aP vaccines, even after 5 doses [19,20], but appears to be more durable after immunization with wP vaccines [22,23] or natural infection in humans [30]. Here we compared the capacity of immunization with aP and wP vaccines versus previous infection to induce T_RM_ cells and to sustain protective immunity in the nose and lung (Figure 4(a)). Assessment of the bacterial load in the respiratory tract 9 months after previous infection revealed sustained protective immunity against nasal colonization and lung infection. The bacterial counts in the lungs and nose were significantly reduced on day 3 and cleared 7 days post challenge (Figure 4(b)). The bacterial load in the lung and nose was also significantly reduced in mice immunized with a wP vaccine, though long-term protection was not as effective as that induced by natural infection, suggesting that there is some waning of immunity after immunization with the wP vaccine. Protective immunity against lung infection induced by immunization with the aP vaccine had also waned after 7 months, and consistent with the earlier experiments (Figure 1(a)), immunization with the aP vaccine did not prevent nasal colonization (Figure 4(b)).

Assessment of the persistence of T_RM_ cells in the respiratory tissue revealed a high number of CD69^+^CD4^+^ T_RM_ cells in the lungs and nasal tissue 7 days after secondary infection with *B. pertussis*. CD69^+^CD4^+^ T_RM_ cells were also expanded in the lung and nasal tissue after challenge in wP-immunized mice, but not in aP-immunized mice, where they were at background levels similar to thse in the non-immunized control mice. Furthermore, natural infection or immunization with the wP vaccine induced CD69^+^CD4^+^ T_RM_ cells that secreted IL-17, IFN-γ or both cytokines, indicative of polyfunctional memory T cells. In contrast, CD69^+^CD4^+^ T_RM_ cells from aP-immunized mice were at background levels, similar to non-immunized control mice 7 days after *B. pertussis* challenge. Interestingly, the frequency and absolute numbers of IL-17-secreting CD69^+^CD4^+^ T_RM_ cells in the lungs and nasal tissue were significantly higher in mice that had previously been infected when compared with mice immunized with the wP vaccine (Figure 4(c,d)). The absolute numbers of IFN-γ-secreting CD69^+^CD4^+^ T_RM_ cells were not significantly different in previously infected and wP-immunized mice, but were significantly higher in mice immunized with the wP compared with the aP vaccine. We examined the correlation between the number of T_RM_ cells in the nasal tissue or lungs and protection, expressed as the ratio of the area under the bacterial clearance curve for immunized or previously infected over that of unimmunized control mice. The results revealed a highly significant correlation between the number of IL-17- or IFN-γ-secreting CD69^+^CD4^+^ T_RM_ cells and long-term protection against nasal colonization (Figure 4(e), supplementary Figure 2(a)). We also found a significant correlation between the number of IL-17-secreting, but not IFN-γ-secreting, CD69^+^CD4^+^ T_RM_ cells and long-term protection against lung infection (supplemental Figure 2(b)).

### Treatment of mice with FTY720 during immunization with a wP vaccine attenuates protective immunity

**Figure 5.**
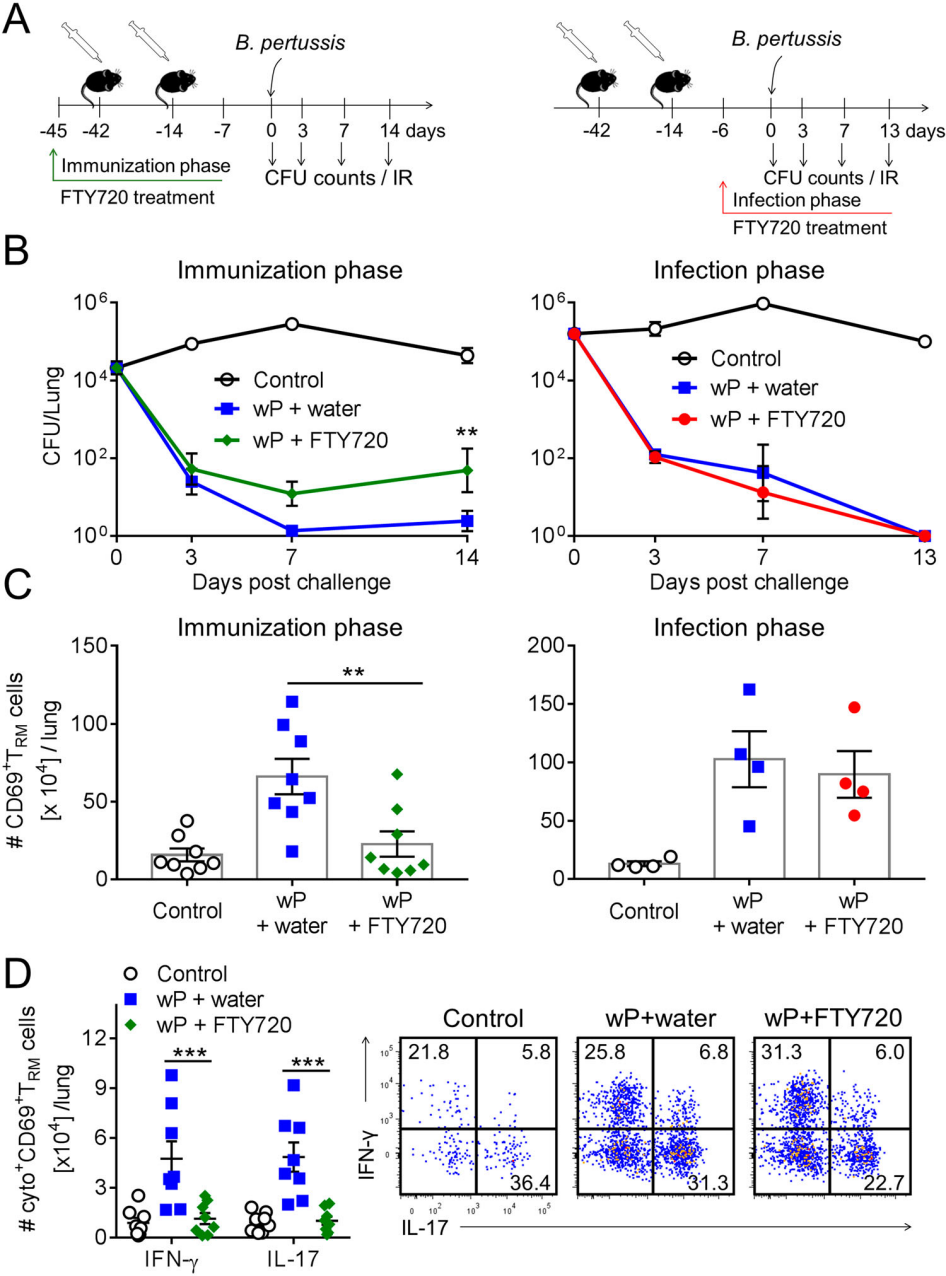
Preventing lymphocyte migration from lymph nodes during wP immunization suppresses accumulation of CD4 T_RM_ cells in the lungs and impairs protection. (a) Schematic of experimental plan; C57BL/6 mice were immunized with a wP vaccine 6 and 2 weeks before challenge with *B. pertussis*. wP-immunized mice were given FTY720 in the drinking water during the immunization phase (3 days before 1st immunization until 7 days after 2nd immunization) or during infection (6 days before *B. pertussis* challenge and during infection). (b) CFU counts in the lungs at intervals after aerosol challenge with *B. pertussis*. Lung CFU counts (left graph: mean ± SEM *n* = 8 mice per group per time-point in two independent experiments; right graph: mean ± SEM *n* = 4 mice per group per time-point). **p < .01 versus wP + FTY720 by two-way ANOVA with the Tukey’s post-test, only significant differences between experimental groups are indicated. Seven days post challenge, mice were injected i.v. with anti-CD45 antibody 10 min prior to euthanasia to discriminate circulating from tissue-resident leukocytes by flow cytometry. (c) Total number of CD69^+^CD4^+^ T_RM_ cells. Results shown are mean ± SEM (left graph: *n* = 8 mice in two independent experiments; right graph: *n* = 4 mice). **p < .01 versus wP + FTY720 by one-way ANOVA with the Tukey’s post-test. (d) Total number of IFN-γ- and IL-17-secreting CD69^+^CD4^+^ T_RM_ cells in the lungs with representative plots showing secretion of IFN-γ and IL-17 by CD45iv^–^CD44^+^CD62L^–^CD69^+^CD4^+^ T cells in wP-immunized mice treated with FTY720 during the immunization phase. Results shown are mean ± SEM (*n* = 8 mice in two independent experiments). ***p < .001 versus wP + FTY720 by two-way ANOVA with the Tukey’s post-test.

We have previously reported that treatment with FTY720, which prevents the migration of lymphocytes from lymph nodes into tissues, impaired clearance of a primary infection with *B. pertussis* [29]. Here we examined the role of T_RM_ cells in immunity induced by immunization with the wP vaccine by treating mice with FTY720 during the immunization phase or before and after *B. pertussis* challenge of wP-immunized mice (Figure 5(a)). We found that treatment of mice with FTY720 continuously from 3 days before the first to 7 days after the second immunization with the wP vaccine impaired the protective immunity induced by the wP vaccine; the CFU counts were significantly higher in FTY720-treated mice (Figure 5(b)). In contrast, protective immunity induced with a wP vaccine was not affected by treatment with FTY720 from day 6 before infection (Figure 5(b)).

We next examined the effect of blocking leukocyte migration during immunization or during *B. pertussis* challenge of wP-immunized mice on accumulation of lymphocytes or CD69^+^CD4^+^ T_RM_ cells in the lungs. Treatment of mice with FTY720 at the time of immunization with a wP vaccine allowed us to assess whether the vaccine was capable of promoting migration of memory CD4 T cells to the lungs to establish respiratory CD69^+^CD4^+^ T_RM_ during the immunization phase, which expand locally following challenge with *B. pertussis*. We found that treatment of mice with FTY720 during immunization with a wP vaccine resulted in a significant reduction in the total number of CD4 T and CD8 T cells infiltrating the lungs (Supplementary Figure 2(a)). It also reduced the number of B cells though not significantly. Administration of FTY720 during the immunization phase also resulted in a significant reduction in the number of CD69^+^CD4^+^ T_RM_ cells in the lung after *B. pertussis* challenge when compared with untreated wP-immunized mice (Figure 5(c)). While the frequency of IFN-γ- or IL-17-secreting CD69^+^CD4^+^ T_RM_ cells was not dramatically affected, the total number of IFN-γ- and IL-17-producing CD69^+^CD4^+^ T_RM_ cells was significantly reduced in FTY720-treated wP-immunized mice (Figure 5(d)). Treatment with FTY720 appeared to reduce the frequencies of IFN-γ^+^IL-17^-^ relative to IFN-γ^–^IL-17^+^ cells (Figure 5(d)). We also assessed the effect of FTY720 on accumulation of T cells in the nasal tissue. However, in these studies parenteral immunization with the wP vaccine did not induce significant accumulation of CD4 T cells in the nasal tissue prior to *B. pertussis* challenge, making it difficult to detect the effect of FTY720.

Mice that had been immunized twice with a wP vaccine and were continuously treated with FTY720 from day 6 before infection showed a significant reduction of total CD8 T cells and B cells in FTY720-treated compared with untreated mice (Supplementary Figure 3(b)), suggesting that these cells temporarily infiltrated the lungs during the infection. The total number of CD4 T cells was also reduced, though not significantly, in FTY720-treated wP-immunized mice (Supplementary Figure 3(b)). The total number of CD69^+^CD4^+^ T_RM_ cells was similar in FTY720-treated and untreated wP-immunized mice (Figure 5(c)). Moreover, the frequency of CD69^+^CD4^+^ T_RM_ cells increased in wP-immunized mice treated with FTY720 when compared with untreated wP-immunized mice (Supplementary Figure 3(c)). This suggests that CD45iv^-^CD69^-^CD4^+^ T cells were not a resident population, but were temporarily infiltrating effector memory T (T_EM_) or effector T (T_eff_) cells. Our findings indicate that the population of lung T_RM_ cells established during the immunization with the wP vaccine is critical for the optimal control of infection in the lower respiratory tract.

### Adoptively transferred lung T_RM_ cells migrated to the lungs, reduced pathogen burden and acquired polyfunctional properties following *B. pertussis* challenge

**Figure 6.**
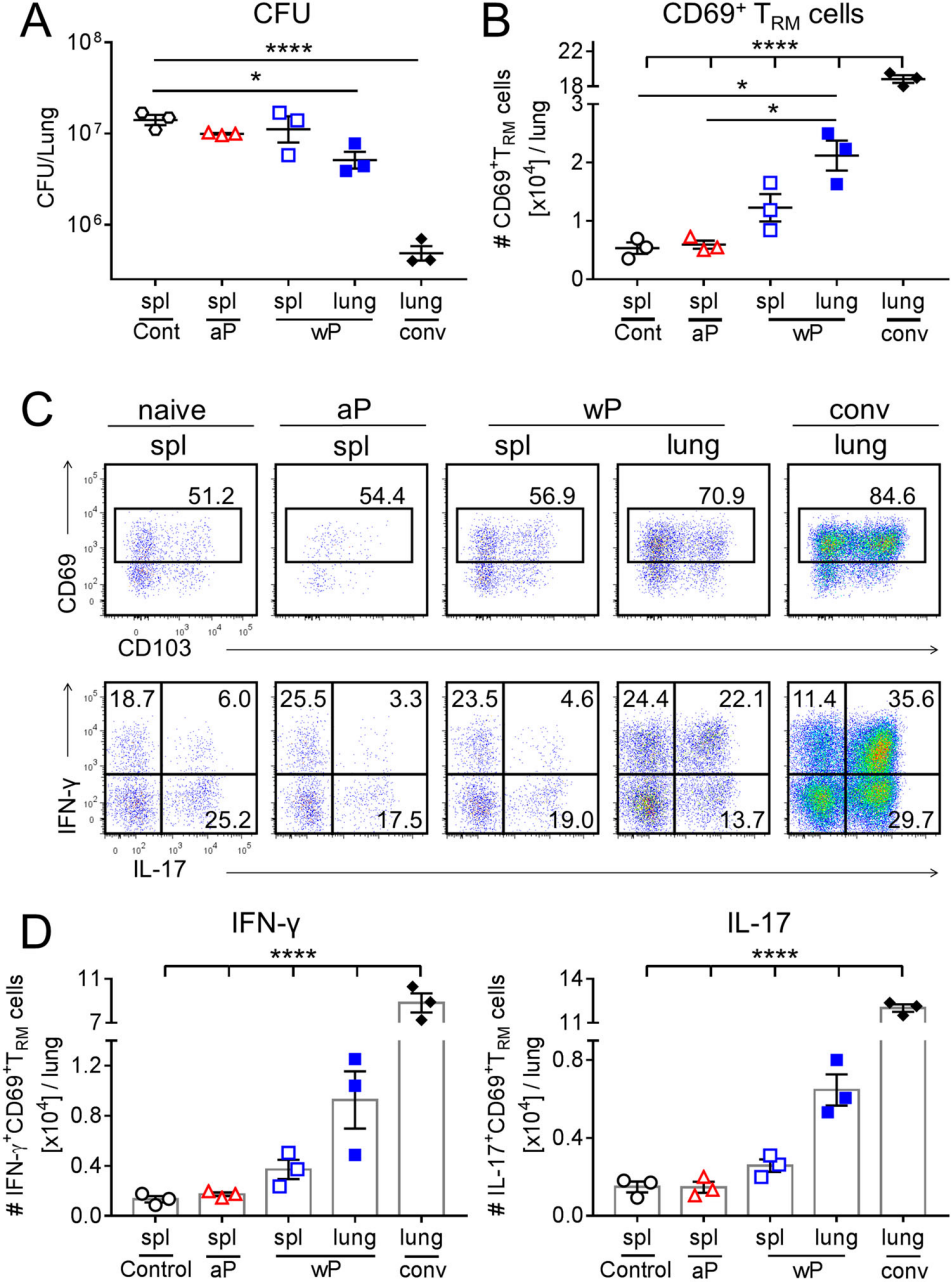
CD69^+^CD4^+^ T_RM_ cells that secrete IFN-γ and IL-17 accumulate in the lungs and reduce bacterial burden in naïve recipient mice after transfer from wP-immunized mice. C57BL/6 mice were immunized with aP and wP vaccines at 6 and 2 weeks or were challenged with *B. pertussis* 7 weeks before euthanasia. Anti-CD45 Ab was administered i.v. 10 min. prior to euthanasia to discriminate circulating from lung resident leukocytes. CD45iv^–^CD4^+^ T cells were purified from the lungs of wP-immunized or convalescent mice and CD4^+^ T cells from the spleens of aP- or wP-immunized or naive control mice. 2 × 10^5^ lung and 4 × 10^5^ spleen CD4 T cells were adoptively transferred to naïve irradiated mice one day prior to aerosol challenged with *B. pertussis*. (a) CFUs in the lungs were enumerated at day 13 post challenge. Results shown are mean ± SEM (*n* = 3 mice). *p < .05; ****p < .0001 by one-way ANOVA with the Tukey’s post-test. (b) Total number of CD69^+^CD4^+^ T_RM_ cells in the lungs at day 13 post challenge (c) Flow cytometry analysis showing representative plots of CD69 and CD103 expression on CD45iv^–^CD44^+^CD62L^–^CD4^+^ T cells and IFN-γ and IL-17 secretion by CD45iv^–^CD44^+^CD62L^–^CD69^+^CD4^+^ T_RM_ cells and (d) total number of IFN-γ- and IL-17-secreting CD69^+^CD4^+^ T_RM_ cells in the lungs at day 13 post challenge. Results shown are mean ± SEM (*n* = 3 mice). ****p < .0001 by one-way ANOVA with the Tukey’s post-test.

To further address the question of whether T_RM_ cells induced with a wP vaccine mediate protection against *B. pertussis* infection, we assessed the capacity of purified lung tissue-resident CD4 T cells or splenic CD4 T cells from immunized mice to transfer protection to naïve mice. As a comparison, we also transferred lung tissue-resident CD4 T cells from convalescent mice. Naive mice or mice immunized with an aP vaccine did not have sufficient T cells in the lung to include these additional experimental groups. Therefore, we assessed the protective effect of splenic CD4 T cells from naïve, aP- or wP-immunized mice. The lung-resident CD4 T cells (2 × 10^5^ cells/mouse; CD45iv^–^ after administering anti-CD45 i.v. before sacrifice) or splenic CD4 T cells (4 × 10^5^ cells/mouse) were transferred into naïve irradiated mice one day before challenge with *B. pertussis* and CFU counts were determined in the lungs 13 days post challenge. Transfer of tissue-resident CD4 T cells from convalescent mice significantly reduced the CFU counts by about 45-fold compared with control infected mice (Figure 6(a)). Mice that received CD4 T cells from the lungs of wP-immunized mice also had significantly reduced bacterial burden when compared with control mice that received spleen CD4 T cells from unvaccinated mice, despite the fact that only 2 × 10^5^ cells were transferred to each recipient mouse (Figure 6(a)). In contrast, transfer of spleen cells from aP- or wP-immunized mice did not significantly reduce the bacterial burden.

We examined the migration of transferred T cells using the intravenous labelling approach and found that transfer of lung CD45iv^–^CD4 T cells resulted in accumulation of CD4 T cells in the lungs of recipient mice 13 days post challenge. The frequency and absolute numbers of CD69^+^CD4^+^ T_RM_ cells were significantly elevated in the lungs of mice that received lung T cells from convalescent or wP-immunized mice, when compared with mice that received splenic CD4 T cells from wP- or aP-immunized mice (Figure 6(b,c)).

To determine whether adoptive transfer of cells to naïve mice enhanced the Th1 and Th17 responses in the lungs after infection, we assessed IFN-γ and IL-17 production by ICS and flow cytometry on lung CD69^+^CD4^+^ T_RM_ cells 13 days post challenge. A significant number of the T cells in the lungs of mice that received lung T cells from convalescent or wP-immunized mice had polyfunctional phenotypes, robustly co-secreting both IFN-γ and IL-17 (Figure 6(c)). Furthermore, there was a higher number of IFN-γ- and IL-17-secreting CD69^+^CD4^+^ T_RM_ cells in recipient mice transferred with lung CD4 T cells from convalescent or wP-immunized mice compared with mice that had received splenic CD4 T cells from aP- or wP-immunized mice (Figure 6(d)). Our data demonstrate that lung tissue-resident CD4 T cells transferred from convalescent or wP-immunized mice accumulate in the lungs of recipient mice as T_RM_ cells, where they potently secrete IFN-γ- and IL-17 and help to reduce the pathogen burden.

### Induction of respiratory CD4 T_RM_ cells, especially those that secrete IL-17, correlates with protection against nasal colonization with *B. pertussis*

**Figure 7.**
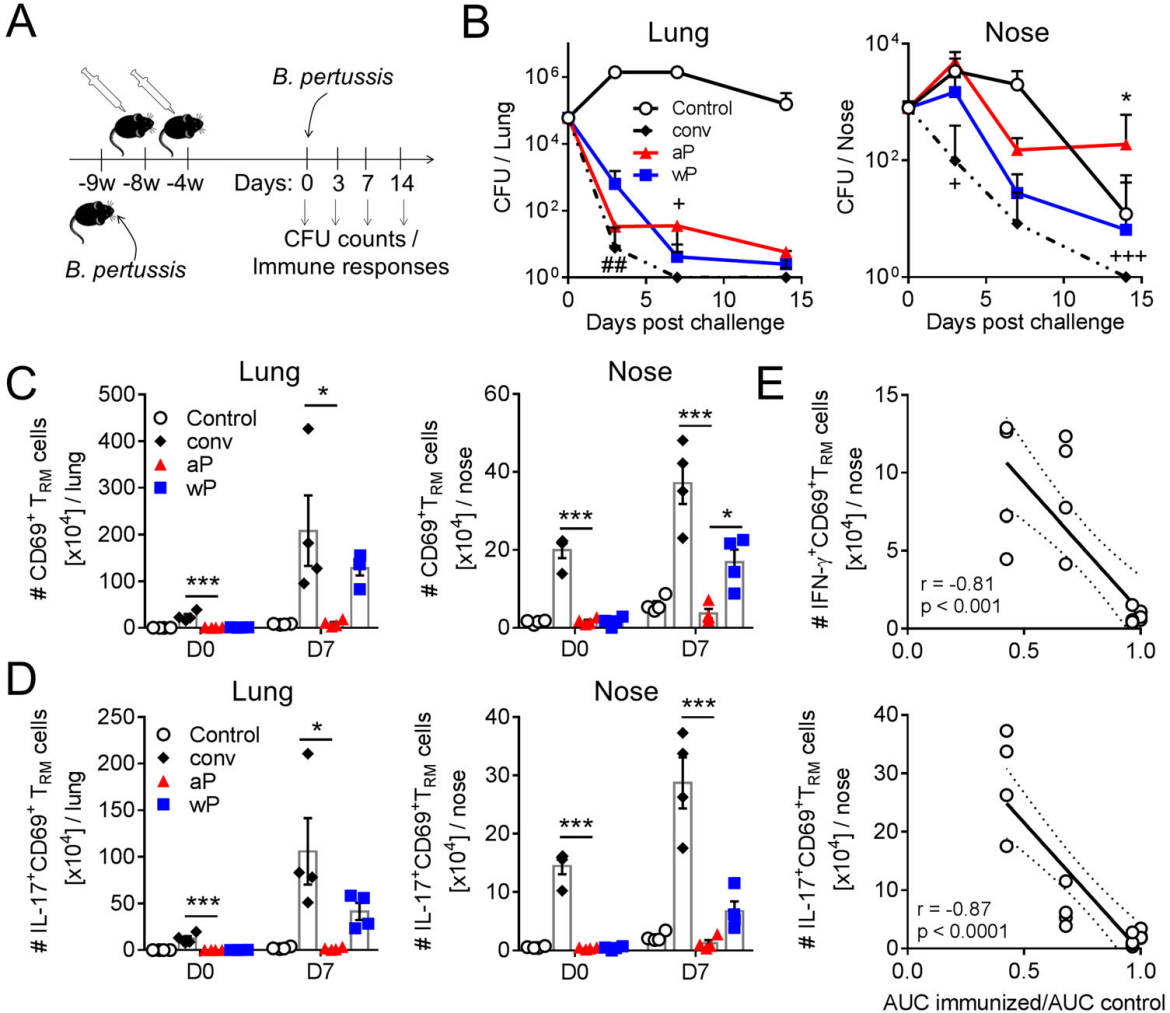
Respiratory CD4 T_RM_ correlate with protection against nasal colonization with *B. pertussis*. (a) Schematic of immunization and challenge protocol. C57BL/6 mice were either aerosol infected with *B. pertussis* (conv), or immunized with 2 doses of either aP (Infanrix) or wP (Shan 5) at an interval of 4 weeks. 9 weeks after challenge and 4 weeks after the second immunization, mice were aerosol challenged with *B. pertussis.* (b) CFUs in lung homogenates and nasal washes were determined 3, 7, and 14 days post challenge. Results are mean ± SEM *n* = 4 mice per group per time-point. *p < .05 aP versus wP; ^##^*p* < .01 wP versus conv; ^+^*p* < .05, ^+++^*p* < .001 aP versus conv by two-way ANOVA with the Tukey’s post-test. One day before challenge (pre) and 7 days post challenge (d7) lymphocytes from the three right lobes of the lung and nasal tissue were prepared by digest. Prior to euthanasia, the animals were i.v. injected with fluorochrome-coupled CD45 antibody to allow the discrimination of tissue-resident and circulating cells. (c) Absolute numbers of CD69^+^CD4^+^ T_RM_ cells in the lung or nasal tissue. (d) Absolute numbers of IL-17-producing CD69^+^CD4^+^ T_RM_ cells in the lung or nasal tissue. Results shown are mean ± SEM (*n* = 4 mice). One-way ANOVA for immunized groups, *p < .05, ***p < .001, only significant differences in comparison to aP are indicated. (e) Correlation between protection against nasal colonization and the absolute number of IFN-γ- or IL-17-producing CD69^+^CD4^+^ T_RM_ in the nasal tissue for the corresponding mouse. Protection was expressed as a ratio of the area under the curve (AUC) of bacterial clearance (day 0–14) after challenge of immunized or previously infected versus the AUC for control unimmunized mice, as previously described [45]. Dotted lines indicate 95% confidence bands of the best fit line. *N* = 4 mice/group at each time point.

Our study has revealed that immunization of mice with a wP vaccine prevented nasal colonization and primed CD69^+^CD4^+^ T_RM_ cells, whereas an aP vaccine failed to protect against nasal colonization or to induce respiratory T_RM_ cells. In order to demonstrate that these novel findings were not an unusual feature of the specific aP (Boostrix) and wP (NIBSC) vaccines that we had used, we extended and confirmed our findings using alternative wP and aP vaccines, which had different compositions to those in the early part of the study. We used the wP-containing vaccine, Shan-5, which is used for paediatric immunization in many developing countries including India and an aP-containing vaccine, Infanrix, which is used for paediatric immunization in developed countries. Infanrix has a higher antigen content than Boostrix. Shan-5 is alum adsorbed whereas the NIBSC wP vaccine is not. In this experiment, we also included a group of convalescent mice. Mice were either immunized twice with aP (Infanrix) or wP (Shan-5) vaccines at an interval of 4 weeks or challenged with *B. pertussis*. Four weeks after the second immunization or 9 weeks after primary challenge, mice were aerosol challenged or re-challenged with *B. pertussis* (Figure 7(a))*.* Consistent with our earlier data, the results revealed that previous infection induces the highest level of protection against infection with *B. pertussis*; bacteria were completely cleared from the lungs by day 7 post re-infection (Figure 7(b)). Immunization with wP or aP vaccines also induced protection against lung infection, though not as effectively as previous infection (Figure 7(b)). Immunization with the aP vaccine (Infanrix) in this experiment appears to induce more complete protection against lung infection (Figure 7(b)) compared with that induced with Boostrix (Figure 1(a)). This may reflect the higher antigen dose in Infanrix compared with Boostrix, but we would caution against making direct comparison between studies, which had other variables including the time between the last immunization and challenge (2 versus 4 weeks, respectively). In the nose, previous infection also induced the best protection, with complete clearance 14 days after re-infection. Immunization with the wP vaccine also conferred significant protection in the nose, with about 100 fold lower CFU count on day 7 compared with the unimmunized control mice. In contrast, immunization with the aP vaccine did not prevent nasal colonization. Indeed, the CFU counts in the nasal cavity were higher on day 14 in aP-immunized compared with non-immunized control mice (Figure 7(b)). Although this was not observed with Boostrix (Figure 1(a)), it is consistent with the studies in baboons [17], which showed more prolonged nasal colonization after *B. pertussis* challenge of aP-immunized compared with naïve baboons.

We assessed the number of T_RM_ cells on the day of *B. pertussis* challenge and 7 days after challenge. The results revealed that CD69^+^ T_RM_ cells were still present in the respiratory tract, especially the nasal tissue, following clearance of a primary infection with *B. pertussis*; these cells expanded dramatically 7 days after re-infection (Figure 7(c)). On the day of challenge, the number of CD69^+^CD4^+^ T_RM_ cells was close to background level in the nose and lung of immunized mice. However, on day 7 post challenge, the number of CD69^+^CD4^+^ T_RM_ cells was significantly expanded in the lung and nose in wP-immunized, but not in aP-immunized mice. (Figure 7(c)). Assessment of cytokine production by CD69^+^CD4^+^ T_RM_ cells in the lungs and nose revealed that a significant proportion of these cells produced IL-17 (Figure 7(d)) and a smaller number secreted IFN-γ (data not shown). The greatest number of IL-17 or IFN-γ-secreting CD69^+^CD4^+^ T_RM_ cells in the lungs and nose 7 days post challenge was induced by previous infection, followed by wP-immunization, while immunization with an aP vaccine did not induce cytokine production above control level.

Finally, we found a strong correlation between the bacterial clearance from the nose and the number of IFN-γ-secreting CD69^+^CD4^+^ T_RM_ cells in the nasal tissue (Figure 7(e)). However, the strongest correlation with protection in the nose was with IL-17-secreting CD69^+^CD4^+^ T_RM_ cells (Figure 7(e)). There was a less significant correlation between protection against lung infection and the number of IL-17-secreting CD69^+^CD4^+^ T_RM_ cells in the lungs (Supplemental Figure 4(a)). Together, with our data involving treatment with FTY720 or T cell transfer, these findings suggest that CD69^+^CD4^+^ T_RM_ cells, especially those that secrete IL-17, play a critical role in adaptive protective immunity against *B. pertussis* locally in the upper and lower respiratory tract.

## Discussion

It is generally accepted that previous infection or immunization with wP vaccines confers more complete and sustained protective immunity against *B. pertussis* infection than immunization with aP vaccines [22,23,30]. Studies in immunized children have reported that antibody responses and protective immunity wane rapidly after immunization with aP vaccines [19,31]. This may reflect poor induction of memory T and/or B cells. The significant new findings of this study are that immunization of mice with wP, but not aP, vaccines primes respiratory T_RM_ cells that rapidly expand in the lung and nasal cavity after infection with *B. pertussis* and mediate rapid bacterial clearance from the lungs and nose. Furthermore, our studies in the mouse model showed that immunization with an aP vaccine protected against lung infection, but failed to prevent nasal colonization, whereas immunization with a wP vaccine prevented infection in the lungs and nose. However, the most persistent protection was induced by natural infection and this reflected potent induction of T_RM_ cells. Our findings support the suggestions that the resurgence of whooping cough may reflect failure of the aP vaccine to prevent nasal colonization and transmission of *B. pertussis* [17,18] or to induce long-term protective immunity [22], due to poor induction of memory T cells, especially those in the respiratory tissues.

aP vaccines were introduced in the 1990s and 2000s as a safer alternative to wP vaccines which were reactogenic, and therefore associated with many unacceptable side-effects. While aP vaccination programmes have been effective in maintaining a low level of pertussis disease in many countries, there have been recent epidemics and infant deaths from whooping cough in several countries using aP vaccines, including the US, UK, Ireland, Australia and the Netherlands [2]. Maternal immunization studies have reported that aP vaccination of pregnant women is highly effective at preventing deaths from pertussis in infants in the first months of life [7]. This appears to be mediated by maternal antibodies specific for PT and is consistent with the view that aP vaccines prevent pertussis disease through induction of circulating antibodies against the protective antigen in the aP vaccines [32,33]. Indeed recent studies in the baboon model have demonstrated that infants born to mothers immunized with a monocomponent pertussis toxoid vaccine were protected against disease, but not against nasal colonization, after challenge with *B. pertussis* [34].

Earlier studies in baboons have demonstrated that while previous infection prevented nasal colonization following re-infection with *B. pertussis,* and immunization with a wP vaccine reduced the CFU counts in the nasopharynx, immunization with an aP vaccine did not prevent nasal colonization or transmission of *B. pertussis* to naïve baboons [17]. While mice cannot be used for *B. pertussis* transmission studies because they do not cough, our results in the mouse model of nasal colonization are entirely consistent with the findings of Merkel and colleagues in the baboon model. The phase 3 clinical trials in children carried out in the 1990s demonstrated that various aP vaccines prevent severe disease [8,9], however, these studies did not assess the capacity of the vaccines to prevent infection or transmission of *B. pertussis*. Subsequent studies have suggested that aP vaccination may not prevent asymptomatic transmission of *B. pertussis* in humans [18]. Collectively, the animal model data provide firm experimental evidence to support these clinical studies which have suggested that aP vaccines do not prevent nasal colonization with *B. pertussis* in humans. So a major question that now needs to be addressed is what is the mechanism of immune protection in the nasal cavity and how can we induce the appropriate immune responses that prevent nasal colonization.

There is convincing evidence from mouse models that cellular immune responses, especially Th1 cells induced by previous infection or immunization with wP vaccines, mediate protection against lung infection with *B. pertussis* [35,36]. IFN-γ secreted by Th1 cells and NK cells activates macrophages and promotes opsonizing antibody production, which helps to clear *B. pertussis* from the respiratory tract [16,37]. There is also growing evidence of a role for Th17 cells in protection against *B. pertussis* through activating neutrophils [15]. Previous studies in baboon and mouse models, and in humans, have indicated that infection with *B. pertussis* or immunization with a wP vaccine induces Th1 and Th17 cells, whereas aP vaccines preferentially induce Th2 cells [13,15,17,36]. This is consistent with the observations that aP vaccines induce potent antibody responses, however, these antibody responses wane relatively quickly after immunization with aP vaccines [22,31,38]. In contrast, studies in mice and humans have shown that cellular immune responses are more persistent after immunization with wP vaccines [25,39]. Interestingly, immunization with the aP vaccine was associated with a reduction in the frequency of IFN-γ-secreting and IL-17-secreting T cells in the lung post *B. pertussis* challenge. Furthermore, consistent with studies in the baboon model [17], we found more prolonged nasal colonization after the aerosol challenge of mice immunized with Infanrix compared with non-immunized control mice. It is possible that the polarized Th2-type responses in aP-immunized mice may suppress the induction of T_RM_ cells and clearance of bacteria from the nasal tract after *B. pertussis* challenge.

Most of the studies on cellular immune responses to *B. pertussis* have focused on systemic T cell responses, based on peripheral blood samples from humans or baboons, and spleen and lymph node cells in mice. While these studies have provided valuable data on the function of Th1 and Th17 cells, they may not reflect the local immune responses at the site of infection in the respiratory tract. We have developed the methodology to examine local immune responses in the nose and have found that there is significant infiltration of immune cells, especially T cells, into the nasal tissue during infection with *B. pertussis*. The results of the present study reveal dramatic differences between aP and wP vaccines in their ability to promote cellular infiltration in the nasal tissue and lungs following infection with *B. pertussis*. Recent reports on other mucosal pathogens have suggested that memory T cells that reside in tissue, termed T_RM_ cells, mediate long-term protection against re-infection at the same mucosal site with the same pathogen [26–28]. Indeed we have already reported that CD69^+^CD4^+^ T_RM_ cells accumulate in the lungs during infection with *B. pertussis*, expand after re-infection and promote clearance of the secondary infection [29]. In this study, we demonstrated that T_RM_ cells also accumulate in the nasal tissue during infection with *B. pertussis* and expand after re-infection. We also found that CD69^+^CD4^+^ T_RM_ cells are expanded in the lungs and nasal tissue after challenge of mice immunized with a wP vaccine. The adoptive transfer experiments showed that lung tissue-resident CD4 T cells home back to the lung and help to mediate bacterial clearance. Furthermore, transfer of small numbers of CD4 T cells from the lungs of convalescent or wP-immunized, but not from the spleen of wP- or aP-immunized mice, conferred some protection against infection of naïve mice with *B. pertussis*. The effect was more striking with lung tissue-resident CD4 T cells from convalescent mice, which is consistent with the view that T_RM_ cells are more effectively generated by infection or immunization at mucosal surfaces [28,40,41]. In contrast, immunization with an aP vaccine completely failed to promote accumulation of CD69^+^CD4^+^ T_RM_ cells in the upper or lower respiratory tissues after challenge with *B. pertussis*.

We have recently demonstrated that it is possible to induce respiratory T_RM_ cells in mice with an experimental aP vaccine delivered by the intranasal (i.n.) route and formulated with a more potent adjuvant than alum [41]. We found that i.n. immunization induced respiratory CD69^+^CD4^+^ T_RM_ cells that were detectable in the lungs and nasal tissue prior to aerosol challenge with live *B. pertussis*. The translation of our findings to humans will not be straightforward. In the present study, we used the i.p. route for parenteral immunization and although not directly translatable to humans, we have previously reported that similar immune response are induced following immunization of mice with pertussis vaccines by i.p. and s.c. routes [42]. Lack of routine access to human respiratory mucosal tissue is also an obstacle to routine detection and quantification of respiratory T_RM_ cells in humans. However, it may be possible to address this, at least on a small scale, using bronchoalveolar lavage or nasopharyngeal aspirates.

Further evidence of a protective role for respiratory CD69^+^CD4^+^ T_RM_ cells was provided by the observation that blocking migration of T cells to the respiratory tissue with FTY720 during immunization with the wP vaccine reduced the protective effects of the vaccine. The CD4 T_RM_ cells induced by natural infection or immunization with the wP vaccine secreted Th1- and Th17-type cytokines, especially IL-17. Furthermore, protection against nasal colonization showed a very strong correlation with the number of cytokine-secreting CD69^+^CD4^+^ T_RM_ cells, especially IL-17-secreting CD69^+^CD4^+^ T_RM_ cells. Collectively, our data suggest that CD4^+^ T_RM_ cells play a critical role in long-term protective immunity against *B. pertussis*. Therefore, future immunization strategies to control the spread of *B. pertussis* must consider the induction of respiratory CD4^+^ T_RM_ cells.

## Materials and methods

### Ethics statement

All experiments on mice were performed under licence from the Irish Health Products Regulatory Authority and with approval from the Trinity College Dublin Ethics committee. Mice were maintained according to the regulations of the European Union and the Irish Department of Health and Children.

### Mice

C57BL/6 mice were obtained from Harlan Laboratories U.K. or were bred in house in the Comparative Medicine Unit in Trinity College Dublin. Mice were 6–10 weeks old at the initiation of experiments and housed in a specific pathogen–free facility in the Comparative Medicine Unit, Trinity College Dublin. All animal experiments were conducted in accordance with the recommendations and guidelines and under licenses approved by the Health Products Regulatory Authority of Ireland in accordance and with prior ethical approval from Trinity College Dublin Animal Research Ethics Committee.

### Vaccines and immunization

For most experiments, mice were immunized with 1/50 human dose of a wP vaccine (National Institute of Biological Standards and Control (NIBSC), UK) or an aP vaccine (Boostrix, GlaxoSmithKline). Alternatively, mice were immunized with 1/50 human dose of Shan 5 (Shantha Biotechnics, India) or Infanrix (GlaxoSmithKline). Mice were immunized intraperitoneally (i.p.) with the vaccines twice at 4-week intervals.

### B. pertussis respiratory challenge

Respiratory infection of mice was performed 2 weeks, 1 month or 7 months after the second immunization. Mice were infected by aerosol challenge with a culture of *B. pertussis* (Bp338 strain; 1 × 10^9^ CFU/ml) administered using a nebulizer (PARI TurboBOY SX) over 10 min as described previously [43]. In some experiments, immunized mice were compared with convalescent mice; convalescent mice were defined as mice that were >60 d post *B. pertussis* challenge on the day of the second dose of vaccine for immunized mice. The course of infection (or reinfection) was followed by performing CFU counts on lung and nasal homogenates or nasal washes at intervals after challenge as previously described [41].

### Blocking T cell migration with FTY720

FTY720 (Santa Cruz Biotechnology) was continuously administered to mice orally in the drinking water at a concentration of 0.3 mg/kg/d for a period of 3 days before the first immunization and until 7 days after the second immunization (immunization phase) or from 6 days before *B. pertussis* challenge until the cessation of the experiment (infection phase).

### Isolation of lung and nasal tissues

Three lung lobes (superior, middle and inferior) were obtained for flow cytometric analysis and two other lobes (post-caval and left) were harvested to determine CFU counts. The nose tip was dissected and used to assess CFU counts, and the nasal tissue, including the nasal cavity and nasal turbinates, was isolated as described [26] and used for flow cytometric analysis.

### Detection of respiratory tissue-resident T cells

We employed an established technique to distinguish tissue-resident from circulating CD4 T cells [44]. Briefly, naïve or convalescent mice or mice immunized with aP or wP vaccines were injected with an anti-CD45 antibody (BioLegend) intravenously (i.v.) 10 min prior to euthanasia. Circulating lymphocytes are exposed to the antibody and are labelled CD45iv^+^, whereas tissue-resident cells are “protected” and remain CD45iv^−^. “Tissue-resident CD4 T cells” are defined through the expression of CD4 and lack of *in vivo* labelling of CD45 after i.v. injection of mice with anti-CD45 10 min prior to euthanasia.

### Quantification of T_RM_ cells

Mononuclear cell suspensions were prepared from lung and nasal tissue by mechanical disruption (chopping with a scalpel) followed by enzymatic disruption of tissue for 1 h with Collagenase D (1 mg/mL; Sigma-Aldrich) and DNAse I (20 U/ml; Sigma-Aldrich). Next, lungs or nasal tissues were passed through a 70 µm cell strainer to obtain a single-cell suspension, followed by RBC lysis with ACK buffer. Cells were incubated with LIVE/DEAD Aqua (Invitrogen). The cells were incubated with Fc block (BD Biosciences) (1:50) to block IgG Fc receptors followed by a surface staining with fluorochrome-conjugated anti-mouse antibodies for various markers: CD69 (clone H1.2F3), CD45R/B220 (clone RA3-6B2), CD8 (clone 53-6.7), CD3 (clone 17A2), CD4 (clone RM4-5), CD44 (clone IM7), CD62L (cloneMEL-14), CD45 (clone 30-F11), CD103 (clone M290) from Biolegend or Invitrogen. For detection of intracellular cytokines, cells were stimulated for 4 h with PMA (5 ng/mL; Sigma-Aldrich), ionomycin (500 ng/ml; Sigma-Aldrich) and brefeldin A (5 μg/mL; Sigma-Aldrich). Then cells were fixed, permeabilized and stained using eBioscience^TM^ Foxp3/Transcription Factor Staining Buffer Set (ThermoFisher Scientific). Anti-mouse antibodies for IFN-y (XMG1.2) and IL-17 (clone TC11-18H10) were used. Fluorescence minus one were used as controls. Flow cytometric analysis was performed on an LSR Fortessa, and data were acquired using Diva software (BD Biosciences). The results were analysed using FlowJo software (TreeStar). Tissue-resident T cells (defined through lack of *in vivo* labelling with anti-CD45 as described above) that are CD44^+^CD62L^-^ and express CD69, with or without CD103, are considered to be “tissue-resident memory T cells”. The frequencies of CD69^+^CD4^+^ T_RM_ cells or IL-17 or IFN-γ-secreting T_RM_ cells were quantified by flow cytometry after gating on live cells, CD3, CD4, CD45iv^−^, CD44 and CD69. The gating strategies for IFN-γ^+^ and/or IL-17^+^ T_RM_ cells are shown in Supplemental Figure 5. The total number of CD69^+^CD4^+^ T_RM_ cells or IL-17- or IFN-γ-secreting CD69^+^CD4^+^ T_RM_ cells were calculated from the frequency of these cells multiplied by the absolute number of immune cells in the corresponding lung or nasal tissue sample which was determined by counting total live cells in a haemocytometer.

### B. pertussis-specific T cell responses in lung and spleen

Spleen cells from immunized or previously infected mice were stimulated with FHA, sBp or medium only at a concentration of 2 × 10^6^/mL. After 3 days of culture the concentrations of IFN-γ, IL-17 and IL-5 in supernatants were quantified by ELISA. Alternatively, CD45iv^-^ CD4^+^ T cells were purified from the lungs and stimulated (1 × 10^5^/mL) with sBp in the presence of irradiated splenic APC (2 × 10^6^/mL). After 3 days of culture, the concentrations of IFN-γ and IL-17 in supernatants were quantified by ELISA.

### Adoptive transfer of respiratory and splenic CD4 T cells

CD4 T cells were purified from lungs of convalescent mice (60 days post challenge) or mice immunized with a wP vaccine (14 days after the second dose of immunizations) or from the spleens of naïve mice or mice immunized with aP or wP vaccines (14 days after the second dose of immunization). Note: there were insufficient T cells in the lungs of naïve or aP-immunized mice to perform adoptive transfer experiments from the lungs of these mice. Convalescent mice or wP-immunized mice were injected with an anti-CD45 antibody (BioLegend) i.v. 10 min prior to euthanasia. Lung mononuclear cells were pooled from 20 wP-immunized and 20 convalescent mice and prepared as described above, followed by cell separation over continuous 40% Percoll gradient (GE Healthcare). CD45iv^-^ CD4 T cells were sorted using BD FACSAriaII sorter (BD Biosciences). Spleens from 10 naïve, aP- and wP-immunized mice were pressed through a 70-μm cell strainer to obtain a single-cell suspension. CD4 T cells were sorted by negative selection (Miltenyi Biotec). Naïve mice were irradiated (4.5 Gy) 1 d before cell transfer using a Gammacell irradiator. A total of 4 × 10^5^ splenic CD4 cells and 2 × 10^5^ lung CD45iv^–^CD4^+^ T cells were transferred i.v. 1 d before challenge. PBS-injected mice served as controls.

### Statistical analysis

Statistical analysis was carried out using GraphPad Prism 7. One or two-way analysis of variance (ANOVA) followed by the Tukey’s multiple comparison test was used to analyse the statistical significance between three or more groups. *P* values less than 0.05 were considered to be statistically significant. Pearson correlation coefficient (Pearson’s r) was used to measure the linear correlation between protection against nasal colonization, expressed as ratio of the area under the curve of bacterial clearance in immunized and un immunized control mice, and the absolute number of IFN-γ- or IL-17-producing CD4 T_RM_ in the nasal tissue; linear regression, dotted lines indicate 95% confidence bands of the best fit line.
